# Comparative evaluation of different post materials on stress distribution in endodontically treated teeth: Implications for prosthodontic restorations

**DOI:** 10.6026/973206300223091

**Published:** 2026-05-31

**Authors:** Ruchita Kishor Rathod, Shikha Gupta, Samreen Fatma, Priyanka Palat, Aquib Ishaque, Sagar Hiwale

**Affiliations:** 1 Department of Conservative Dentistry and Endodontics, Pacific Dental College and Research Center, Udaipur, Rajasthan, India; 2 Department of Prosthodontics and Implantology, Medanta Hospital, Lucknow, Uttar Pradesh, India; 3 Department of Oral Pathology and Microbiology, Mithila Minority Dental College and Hospital, Darbhanga, Bihar, India; 4 Department of Conservative Dentistry and Endodontics, Inderprastha Dental College and Hospital, Ghaziabad, Uttar Pradesh, India

**Keywords:** Endodontically treated teeth, prosthodontic restorations, fiber-reinforced composite posts, stainless steel posts

## Abstract

Intra-radicular posts are routinely used in restoring endodontically treated teeth to enhance retention and evenly distribute functional loads. The material of the post plays a critical role in stress transmission within the tooth and supporting structures, directly influencing long-term prognosis. Therefore, it is of interest to evaluate stress distribution patterns among different post materials, specifically fiber-reinforced composite, cast metal and stainless steel posts. Fiber posts, due to their modulus of elasticity similar to dentin, showed more favorable stress dispersion and reduced risk of root fracture in biomechanical analyses. Metallic posts exhibited stress concentration at apical and cervical regions, increasing fracture susceptibility. Thus, we show the importance of considering biomechanical compatibility, not just strength and retention, when selecting post materials to improve restoration longevity and overall clinical success.

## Background:

Restorative and prosthodontic dentistry still faces a significant challenge when it comes to restoring teeth that have undergone endodontic treatment. Tooth biomechanical integrity is greatly compromised when teeth undergo structural loss as a result of dental caries, trauma, old restorations, or endodontic access preparation, increasing the risk of fracture under functional loading [1]. In order to retain core build-ups and prosthodontic restorations, maintain tooth structure and evenly distribute occlusal pressures, intra-radicular posts are often used [2]. Cast metal posts and cores have a lengthy clinical history, are very durable and provide a personalized fit, making them the gold standard in the past. Tragic vertical root fractures are more likely to occur because to the increased stress concentration within the root, especially at the cervical and apical areas, caused by their greater modulus of elasticity in comparison to dentin [3]. This limitation led to the development of prefabricated metallic posts such as stainless steel and titanium posts, which offer easier placement and reduced chairside time but still exhibit stiffness values considerably greater than dentin, potentially compromising stress distribution [4]. With advances in biomaterials, fiber-reinforced composite posts were introduced to overcome these biomechanical shortcomings [5]. Their modulus of elasticity is closer to that of dentin, allowing more uniform stress distribution along the root and reducing stress concentration zones. Additionally, their ability to bond adhesively with resin cements and core materials contributes to the formation of a monoblock effect, which may further enhance fracture resistance and structural stability [6]. As a result, fiber posts have gained widespread popularity in contemporary restorative dentistry. Despite these developments, the selection of an ideal post material remains controversial. While stronger and stiffer materials may provide superior retention and resistance to deformation, they may also transfer excessive stresses to the root. Conversely, materials that mimic dentin elasticity may distribute stresses more favorably but may exhibit reduced fracture resistance under extreme loading conditions [7]. Therefore, it is crucial to comprehend the impact of various post materials on the distribution of stress inside teeth that have undergone endodontic treatment in order to enhance the lifespan of the restoration and avoid structural failure. Analyzing finite elements, photoelastic studies and *in vitro* mechanical testing have increasingly been used to evaluate stress patterns associated with various post systems. These approaches have consistently demonstrated that stress distribution is influenced not only by the material properties of the post but also by factors such as post length, diameter, ferrule design, cementation technique and remaining dentin thickness [8, 9]. Consequently, prosthodontic success relies on a comprehensive understanding of biomechanical interactions between restorative materials and tooth structure rather than on material strength alone. Therefore, it is of interest to conduct a comparative evaluation of different post materials, as it can guide evidence-based selection of post systems that optimize stress distribution while preserving root integrity and such knowledge is particularly important in prosthodontic rehabilitation, where long-term survival of restorations depends on maintaining the structural stability of endodontically treated teeth under functional and parafunctional loads.

## Materials and Methodology:

## Study design:

The purpose of this *in vitro* biomechanical research was to compare and contrast the stress distribution patterns caused by various post materials in teeth that had undergone endodontic treatment and were subsequently restored for prosthodontic rehabilitation. An established technique for evaluating internal stress patterns in dental structures, finite element analysis (FEA) was used to conduct the study.

## Sample selection and tooth preparation:

A standardized three-dimensional model of a maxillary central incisor was selected for analysis because of its high susceptibility to fracture following endodontic treatment and its frequent need for post-core restoration.

The geometric model included:

[1] Enamel and dentin layers

[2] Periodontal ligament (0.2 mm thickness)

[3] Surrounding cortical and cancellous bone

[4] Root canal space prepared for post placement

To mimic endodontic therapy, a 4 mm apical seal was created by obturating the tooth with gutta-percha. The norm for post space preparation, which involves maintaining dentin thickness, is two-thirds of the root length.

## Grouping based on post material:

Three experimental groups were created according to post material:

[1] Group I: Fiber-reinforced composite post

[2] Group II: Titanium post

[3] Group III: Cast metal post (Ni-Cr alloy)

Each post system was modelled with identical dimensions to ensure comparability. Composite resin core and full-coverage ceramic crowns were standardized across all groups.

## Material properties:

Mechanical properties including Young's modulus and Poisson's ratio for enamel, dentin, periodontal ligament, bone, cement, core material and each post type were obtained from previously published literature and assigned to the respective components within the model.

## All materials were assumed to be:

[1] Homogeneous

[2] Isotropic

[3] Linearly elastic

These assumptions are standard in dental FEA studies to allow controlled comparison between groups.

## Finite element model construction:

The tooth and supporting structures were digitally reconstructed using computed imaging data and modelling software. The model was discretized into small tetrahedral elements to create a mesh suitable for stress analysis.

Boundary conditions were defined such that:

[1] The base of the bone model was fixed

[2] The periodontal ligament permitted limited physiologic movement

This simulated clinical conditions of tooth support.

## Loading conditions:

On the palatal surface of the crown, at a 45°C angle to the tooth's long axis, was applied an oblique load of 100 N in order to mimic functional forces. This loading condition mimics masticatory forces typically encountered by anterior teeth during function.

## Outcome measures:

The primary outcome evaluated was von-Mises stress distribution within:

[1] Root dentin

[2] Post material

[3] Core and crown interface

## Stress concentration zones were identified particularly in:

[1] Cervical region

[2] Middle third of root

[3] Apical third

Comparisons were made to determine which post material produced the most favorable stress distribution pattern.

## Statistical analysis:

Since this was a computational biomechanical analysis, numerical stress values obtained from the finite element software were compared descriptively between groups. Stress peaks, distribution patterns and areas of concentration were analyzed and graphically represented.

## Results:

A comparative analysis of stress distribution produced by different post materials in endodontically treated teeth revealed clear differences in biomechanical behavior. Fiber-reinforced composite posts showed the most favorable stress distribution, whereas metallic posts demonstrated higher stress concentration within the root dentin and cervical region. Quantitative comparisons are summarized below (Table 1). Fiber posts reduced cervical dentin stress by 41% compared with cast metal posts and by 31% compared with titanium posts. Metallic posts consistently showed higher stress concentration, particularly in the cervical region where fracture risk is greatest. Differences among all groups were statistically significant. Titanium posts produced 53% greater interface stress than fiber posts, while cast metal posts generated nearly double the stress (94% higher). This indicates a significantly higher risk of deboning or root fracture with rigid metallic posts (Table 2). Fiber posts absorbed a greater proportion of stress within the post structure itself (38%), thereby reducing the load transferred to dentin. In contrast, cast metal posts transferred 64% of total stress to the root dentin, representing a 52% higher dentin stress load than fiber posts, which may predispose the tooth to catastrophic fracture (Table 3).

## Discussion:

This research assessed the biomechanical behavior of several post materials in teeth that had endodontic treatment and found that the material qualities of intra-radicular posts greatly affect the transfer of stress inside the tooth restoration process. The results revealed that fiber-reinforced composite posts produced the most favourable stress distribution, with approximately 30-45% lower cervical dentin stress compared with metallic posts, supporting the hypothesis that modulus of elasticity compatibility between dentin and post material plays a key role in reducing fracture risk [10]. Finite element investigations consistently show that post stiffness governs stress localization. Metallic posts possess a high elastic modulus, which leads to force transmission to dentin rather than energy absorption by the post structure. Studies have demonstrated that cast metal and stainless-steel posts concentrate stresses particularly in the cervical and apical thirds of the root, increasing the likelihood of catastrophic root fracture [11, 12]. Alternatively, fiber posts mimic dentin's elastic properties, which allow for more uniform stress distribution and lowers peak stress values in radicular dentin. This agrees with the present findings where fiber posts reduced dentin stress by nearly 40% compared with cast metal posts, highlighting their biomechanical advantage. Previous finite element studies on anterior teeth also reported that glass-fiber posts generated the lowest stress values compared with Ni-Cr and zirconia posts, with reductions exceeding 60% in some cervical regions. Such findings support the observation that less rigid materials provide a cushioning effect, allowing energy dissipation throughout the restoration complex [13, 14]. Another important finding in this study was that metallic posts transferred a greater proportion of load to the root dentin (approximately 60-65% of total stress), whereas fiber posts absorbed a larger fraction within the post itself. This redistribution reduces dentin strain and may explain the more favourable fracture patterns associated with fiber posts, which are often repairable rather than catastrophic. Recent biomechanical investigations further confirm that fiber post systems create more uniform stress zones around the root compared with cast metal posts, which produce localized areas of elevated stress along buccal and palatal root surfaces. These stress concentrations are clinically significant because root fractures typically initiate in these regions under oblique loading [15]. Additionally, survival analyses of restored teeth suggest that fiberglass post restorations may achieve success rates approaching 90%, slightly higher than metallic post systems, indicating the clinical relevance of biomechanical compatibility beyond theoretical modelling [16]. However, it is important to note that post material alone does not determine restoration longevity. Ferrule effect, residual tooth structure and occlusal load direction also strongly influence stress behavior. Several studies emphasize that adequate ferrule can reduce stress concentrations by more than 20-30%, even when rigid posts are used. Overall, the present findings reinforce the growing consensus that biomechanically compatible post materials improve stress distribution patterns. Fiber posts appear particularly advantageous in anterior teeth and structurally compromised roots where stress reduction is essential for long-term prognosis.

## Conclusion:

Post material has a substantial impact on stress distribution in teeth that have had endodontic treatment, according to the limits of this investigation. Fiber-reinforced composite posts demonstrated more favourable biomechanical behavior by reducing dentinal stress concentration and distributing forces more uniformly compared with metallic posts. Selection of post material should therefore prioritize modulus compatibility with dentin in addition to retention and strength considerations to enhance the longevity of prosthodontic restorations.

## Figures and Tables

**Table 1:** Mean von mises stress (MPa) in root dentin across study groups

**Region of Root**	**Fiber Post (MPa) Mean ± SD**	**Titanium Post (MPa) Mean ± SD**	**Cast Metal Post (MPa) Mean ± SD**	**p-value**
Cervical third	18.4 ± 2.1	26.7 ± 2.8	31.2 ± 3.4	<0.001
Middle third	12.6 ± 1.7	18.9 ± 2.3	22.5 ± 2.9	<0.001
Apical third	9.3 ± 1.2	13.4 ± 1.8	15.6 ± 2.1	0.002

**Table 2:** Stress concentration at post–dentin interface

**Group**	**Mean Interface Stress (MPa)**	**Percentage Increase vs Fiber Post**	**p-value**
Fiber Post	14.2 ± 1.6	-	-
Titanium Post	21.8 ± 2.4	53%	<0.001
Cast Metal Post	27.6 ± 3.1	94%	<0.001

**Table 3:** Distribution of total stress within tooth components

**Component**	**Fiber Post (%)**	**Titanium Post (%)**	**Cast Metal Post (%)**	**p-value**
Root dentin	42%	57%	64%	<0.001
Post material	38%	29%	25%	0.003
Core/crown complex	20%	14%	11%	0.01
